# Long-term survival of implant-based oral rehabilitation following maxillofacial reconstruction with vascularized bone flap

**DOI:** 10.1186/s40729-022-00413-7

**Published:** 2022-04-05

**Authors:** Hongyang Ma, Jeroen Van Dessel, Sohaib Shujaat, Michel Bila, Yi Sun, Constantinus Politis, Reinhilde Jacobs

**Affiliations:** 1 OMFS IMPATH Research Group, Department of Imaging & Pathology, Faculty of Medicine, KU Leuven, Campus Sint-Rafaël, Kapucijnenvoer 33, 3000 Leuven, Belgium; 2 Department of Oral and Maxillofacial Surgery, University Hospitals Leuven, Leuven, Belgium; 3 Department of Dental Medicine, Karolinska Institutet, Stockholm, Sweden

**Keywords:** Dental implants, Head and neck neoplasms, reconstructive surgical procedures, Survival analysis, Free tissue flaps, Radiotherapy

## Abstract

**Aim:**

The aim of the study was to assess the 5-year cumulative survival rate of implant-based dental rehabilitation following maxillofacial reconstruction with a vascularized bone flap and to investigate the potential risk factors which might influence the survival rate.

**Materials and methods:**

A retrospective cohort study was designed. Inclusion criteria involved 18 years old or above patients with the availability of clinical and radiological data and a minimum follow-up 1 year following implant placement. The cumulative survival rate was analyzed by Kaplan–Meier curves and the influential risk factors were assessed using univariate log-rank tests and multivariable Cox-regression analysis.

**Results:**

151 implants were assessed in 40 patients with a mean age of 56.43 ± 15.28 years at the time of implantation. The mean number of implants placed per patient was 3.8 ± 1.3 with a follow-up period of 50.0 ± 32.0 months. The cumulative survival at 1-, 2- and 5-years was 96%, 87%, and 81%. Patients with systemic diseases (HR = 3.75, 95% CI 1.65–8.52; *p* = 0.002), irradiated flap (HR = 2.27, 95% CI 1.00–5.17; *p* = 0.05) and poor oral hygiene (HR = 11.67; 95% CI 4.56–29.88; *p* < 0.0001) were at a significantly higher risk of implant failure.

**Conclusion:**

The cumulative implant survival rate was highest at 1st year followed by 2nd and 5th year, indicating that the risk of implant failure increased over time. Risk indicators that seem to be detrimental to long-term survival include poor oral hygiene, irradiated flap and systemic diseases.

**Supplementary Information:**

The online version contains supplementary material available at 10.1186/s40729-022-00413-7.

## Introduction

The reconstruction of oral and maxillofacial (OMF) defects secondary to tumor, osteonecrosis, trauma, and congenital disease represent a daunting task in head and neck surgery and require a multidisciplinary treatment approach. To this end, vascularized bone flaps serve as the gold standard for OMF reconstruction, which commonly includes, vascularized fibula flap (VFF), deep circumflex iliac artery flap (DCIA), and vascularized osteomyocutaneous scapular flap (VOSF) [1–3]. These flaps benefit from an adequate blood supply, sufficient bone mass and satisfactory flap survival rate [4].

One of the most fundamental parts of the care pathway following maxillofacial reconstruction with a free vascularized bone flap involves oral and maxillofacial rehabilitation for the restoration of facial esthetics, masticatory function, speech, and improvement of the patient’s quality of life [4–6]. The patients undergoing bone flap reconstruction for extensive soft and/or hard tissue loss suffer from insufficient oral vestibular space, stability, and retention capacity, which is a prerequisite for the tissue prosthesis [7–9]. Thereby, dental implant-based rehabilitation acts as the most viable treatment option in such cases.

Previously, several studies have investigated the survival rate of dental implants following vascularized bone flap reconstruction [3]. However, only a few studies exist assessing the cumulative survival rate of implants at a long-term follow-up period of 5 years or more. It is also essential to assess the survival rate based on the functionally loaded implants, for determining whether the patients benefit from implant therapy. At present, differences in survival rate exist among various studies due to the heterogeneity related to the recruitment of patients with a mixture of non-functional (non-restorable or freestanding implants) and functional implants which could impact the overall cumulative survival rate, where patients with functional implants might be associated with a higher risk of implant failure. Hence, requiring further studies to improve the level of evidence at a long-term level.

Furthermore, the association between implant failure and potential risk factors has not been thoroughly investigated. For instance, an increased risk of implant failure has been documented in patients receiving radiotherapy at a dose of 65 Gy and more [10]. Although implant placement after radiotherapy has been suggested to be a relatively safe procedure concerning the long-term impact on peri-implant bone resorption [11]. The impact of radiotherapy on implant survival is seldom reported in relation to its placement in the irradiated bone flap compared to the native bone, thereby, leading to an inadequate representation of the survival rate [12]. Other factors, such as systemic conditions and smoking have also been linked with an increased risk of implant failure, however, lack of evidence exists related to their role on the long-term cumulative survival rate [13]. At the same instance, it is not clear whether the presence of multiple risk factors in a patient could lead to a higher implant failure. Hence, it is important to assess the impact of these risk factors both at an individual and cumulative level.

The primary aim of the study was to determine the 5-year cumulative survival rate of implant-based dental rehabilitation following maxillofacial reconstruction with a vascularized bone flap. The secondary aim focused towards identifying potential risk factors which might contribute towards implant failure.

## Materials and methods

### Patients

A retrospective cohort study was designed following the Strengthening the Reporting of Observational Studies in Epidemiology (STROBE) guidelines [14]. The study was approved by the Medical Ethics Committee of the University Hospitals Leuven, Leuven, Belgium (S-63615) and registered at ClinicalTrials.gov (NCT04884126). The sample consisted of patients who underwent OMF reconstruction at the Department of Oral and Maxillofacial Surgery, University Hospitals Leuven, from December 2004 till January 2020. Inclusion criteria involved 18 years old or above patients with the availability of clinical and radiological data (cone-beam computed tomography (CBCT) or multi-slice CT) and a minimum follow-up 1 year following implant placement. Patients with severe systemic diseases (American Society of Anesthesiologists [ASA] physical status scores of III or more) were excluded [15].

### Reconstructive surgery protocol

Considering the inclusion of 16 years of patients’ records, there were some time-dependent shifts related to the digitalization of the surgical planning protocol. Patients operated before January 2014 were treated with traditional freehand reconstructive surgery and following that time-point onwards computer-assisted surgery (CAS) was performed with either digitalized or non-digitized dental implant surgery. Preoperative CT (slice thickness < 1 mm; Siemens SOMATOM Definition Edge) and CT angiography were acquired for all the patients. As per CAS protocol, the CT images were imported into a three-dimensional (3D) surgical planning software (ProPlan, Version 2.0/3.0 Materialise, Leuven, Belgium) for the generation of maxillofacial models and performing virtual surgery with osteotomy planes. Thereafter, patient-specific surgical guides were designed in a 3D designing software (3-Matic, Version 9.0-13.0, Materialise, Leuven, Belgium). The cutting guides were exported in Standard Tessellation Language (STL) format and printed using a 3D printer (Connex 350, Stratasys, Eden Prairie, MN, USA). Furthermore, the shape, length, number, and size of titanium plates and screws were comprehensively planned according to the planned dental implant position. The reconstructed segmented was either fixated using titanium miniplates and screw system (2 mm non-locking or 2.3 mm locking, KLS Martin Group, Tuttlingen, Germany) or pre-bent reconstructive plates, manually bent on the 3D-printed reconstructed model A fixation tray was used for the guided placement of the reconstructive plates. The screw holes were drilled and osteotomy lines were marked onto the surgical guide. The bone flap was detached from the donor site and modeled according to the templates as planned. Small bony fragments were fixed using screws and plates. Finally, microanastomosis and suturing were performed to close the wound at the recipient site. In patients requiring radiotherapy, it was delivered by a linear accelerator in daily fractions of 2–2.2 Gy five times a week for 6 weeks (60–66 Gy).

### Dental implant placement and prosthetic installation

Prior to implant surgery, all patients were referred to an oral hygienist for achieving an optimal level of oral health. Dental implants were either inserted immediately at the time of surgical reconstruction (Stage I) or delayed placement at ≥ 6 months after grafting (Stage II), depending on the general condition of the patient and administration of adjuvant therapy. The majority of patients who underwent Stage II surgery included the ones who received radiotherapy. The implants were placed in grafted and/or native bone where applicable for ensuring a functional jaw and were inserted at a minimum torque of 35 Ncm using hand ratchet and/or low-speed handpiece. All surgical procedures were performed in compliance with the Brånemark protocol and delayed loading was applied [16]. Before the delivery of the final prosthesis, either a temporary removeable prosthesis or gastrostogavage tube was inserted during the healing phase for the administration of necessary nutrition.

### Postoperative follow-up

Clinical examination was performed once every 6 weeks during the first half-year, every 2 months until the end of the 1st year and every 3 months in the 2nd year. Afterward, the timeframe between the examinations was extended up to 6 months. The overall cumulative survival of dental implants was recorded at the follow-up period of 5 years.

The implants were categorized as “success” or “failure” clinically and radiologically according to the ICOI PISA health scale, where the failure was represented by any of the following: pain on function, mobility, more than 50% radiographic bone loss along the implant length and uncontrolled exudate. Non-restorable (sleepers), exfoliated or surgically removed implants were also categorized as a failure.

**Fig. 1:**
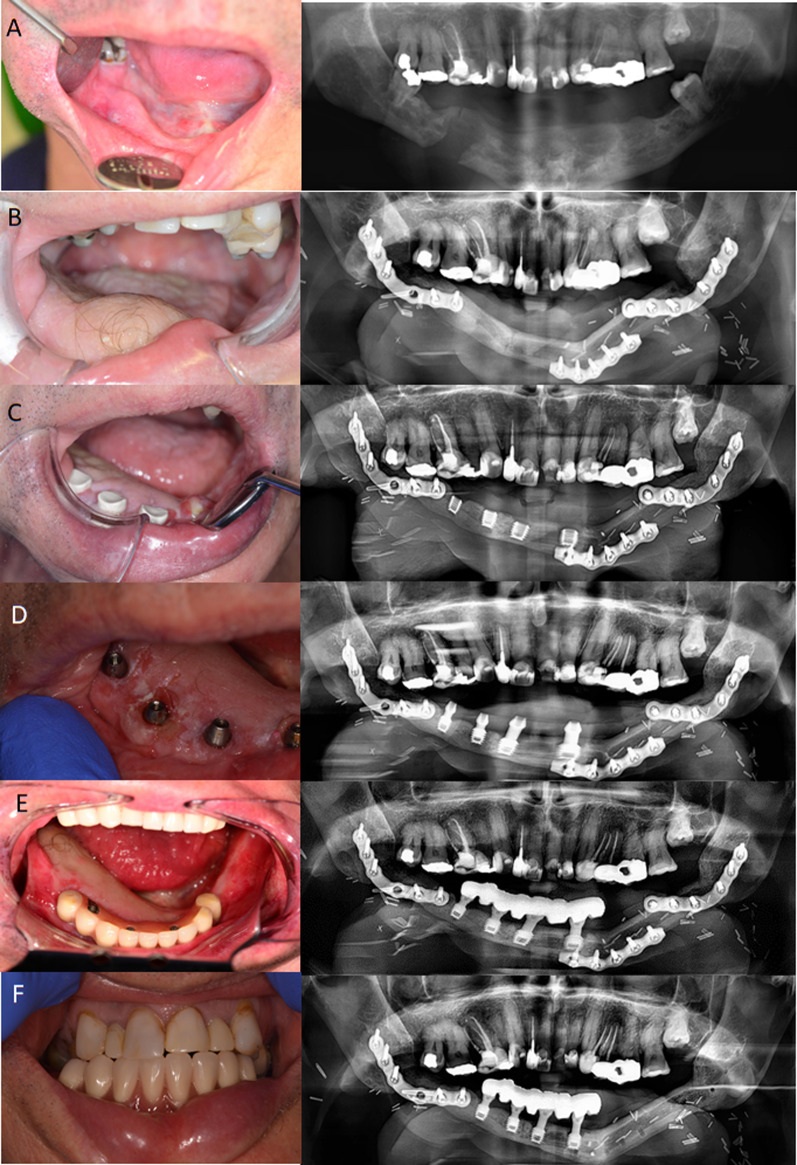
Clinical photos and panoramic radiographs of a 60 6-year-old male patient diagnosed with mandibular osteoradionecrosis with mandibular reconstruction. **A** Intraoral photo and panoramic radiography before reconstructive surgery; **B** intraoral photo and panoramic radiography after mandible reconstruction; **C** intraoral photo and panoramic radiography after dental implant placement; **D** the stability of inserted implants were well after 6 months and implant abutments were installed; **E** fitting restorations are stable in situ after superstructure and dentures installment; **F** a stable occlusal relationship was confirmed after 5 years follow-up

Implant survival was defined as “the implant remaining in situ at follow-up examination” with either satisfactory or compromised status. Satisfactory survival indicated less than ideal conditions, however clinical management was not required. It was represented by absence of pain on function, no mobility, no exudate history and 2 to 4 mm of radiographic bone loss. On the contrary, compromised survival referred to implants requiring clinical management to avoid implant failure and involved, no mobility, absence or presence of sensitivity on function and exudate, radiographic bone loss of > 4 mm (less than one-half length of the implant body) and probing depth of > 7 mm [17]. Figure 1 illustrates an example of a case with clinical and radiographic follow-up after reconstructive and dental implant surgery.

### Study variables

The recorded parameters included age, gender, smoking, primary etiology (malignant tumor, benign tumor or cyst, osteoradionecrosis, trauma), defect size, flap type (fibula, iliac, scapula), flap complications, radiotherapy, implant insertion site (mandible, maxilla/bone flap, native bone), implant insertion stage (stage I, stage II), implant length (≤ 8 mm, > 8 mm), poor oral hygiene (characterized by distinct bleeding gums, dry mouth, bad breath, gum disease, tooth decay, and erosion) and presence of systemic disease (diabetes mellitus, cardiovascular diseases). The defect size was classified based on Brown’s classification, where, a small-sized defect was defined as “Class I” or “Class II”, and large defects included “Class III”, or “Class IV” [18, 19].

### Statistical analysis

Data were analyzed using IBM SPSS Statistics version 25.0 (IBM Corp., Armonk, NY: IBM Corp, USA) and STATA 14.0 (STATA Corp., College, TX, USA). A time-point of 5 years following implant placement was selected as the censored time for cumulative survival analysis. The Kaplan–Meier curves were used to estimate the implant survival rate and the potential risk factors were compared through log-rank tests. The risk factors with a significant *p*-value of < 0.1 based on the univariate log-rank tests were entered into a multivariable Cox-regression analysis for controlling the confounding factors and satisfying the assumptions of a proportional hazard model. Hazard ratio (HR) and 2-sided 95% confidence intervals (CI) for each factor were calculated. A *p*-value of < 0.05 was considered significant.

## Results

### Patient characteristics

**Fig. 2:**
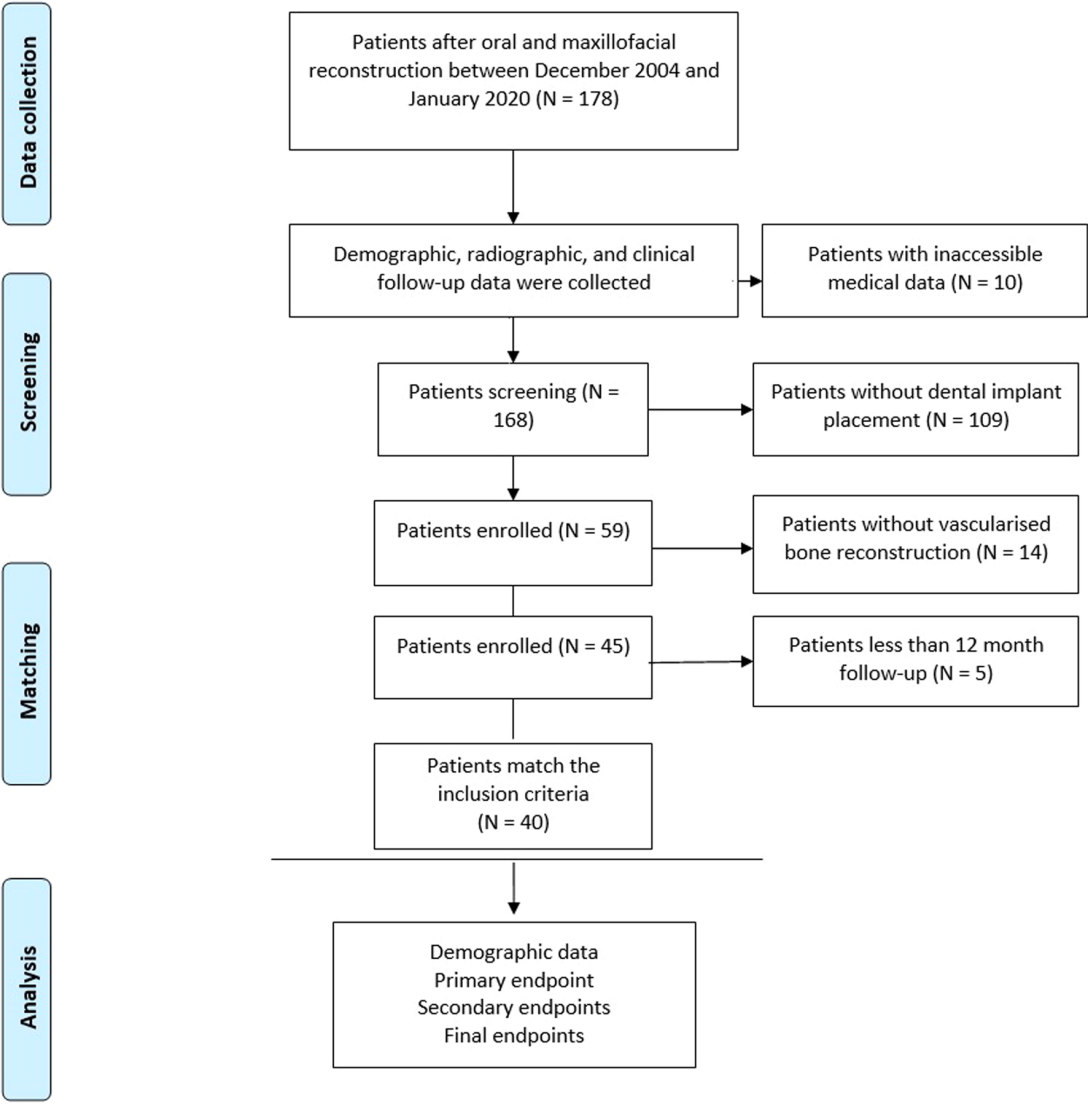
Flowchart of the included patients

Of the data collected from 178 consecutive patients, 138 were excluded based on the following reasons: lack of patient data (*n* = 10), no insertion of dental implant (*n* = 109), patients without vascularized bone flap (*n* = 14), and a follow-up period of less than 12 months following implant placement (*n* = 5) (Fig. 2). The final sample consisted of 40 patients (male: 26, female: 14) with a mean age of 56.43 ± 15.28 years at the time of implantation. The majority of patients were male (65%) and active smokers (65%). Twenty-two patients were diagnosed with a malignant tumor, 5 with benign tumor or jaw cyst, 9 with osteoradionecrosis, and 4 with oral and maxillofacial trauma. Mandibular reconstruction was performed in 35 patients and 5 patients underwent maxillary reconstruction. A vascularized fibular bone flap was used in 31 patients followed by 9 vascularized iliac or scapular flaps (Table 1).

**Table 1:** Patient characteristics

Characteristics	Subgroups	NP (*N*)	NF (*N*)	NI (*N*)	NIF (*N*)
Age (year)	Mean age	56.43 ± 15.28			
	Age range	18–85			
Age	≥ 65	10	6	31	9
	< 65	30	9	120	19
Gender	Male	26	7	100	16
	Female	14	8	51	12
Smoker	Yes	26	13	100	25
	No	14	2	51	3
Etiology	Malignant tumor	22	10	83	17
	Benign tumor or jaw cyst	5	0	16	1
	ORN	9	4	38	9
	Trauma	4	1	14	1
Site	Mandible	35	13	130	26
	Maxilla	5	2	21	2
Flap type	VFF	31	11	131	27
	VIF/scapula	9	4	20	1
IF	Yes	15	8	53	17
	No	25	7	98	11
DIIS	I stage	10	5	35	11
	II stage	30	10	116	17
Implant location	Graft bone	32	12	133	26
	Native bone included	8	3	18	2
Oral hygiene	Good	27	4	110	6
	Poor	13	11	41	22
Flap complication	Yes	12	6	45	13
	No	28	9	106	15
Implant length	> 8 mm	34	11	122	21
	≤ 8 mm	6	4	29	7
Systemic disease	Yes	9	4	34	18
	No	31	11	117	10

*IF* irradiated flap, *ORN* osteoradionecrosis, *VFF* vascularized fibular flap, *VIF* vascularized iliac flap, *VOSF* vascularized osteomyocutaneous scapular flap, *DIIS* dental implant insertion stage, *NP* numbers of patients who received dental implant(s), *FP* numbers of patients with failed dental implant(s), *NI* numbers of implants, *NIF* numbers of implant failure

A total of 151 implants were inserted in 40 patients (vascularized bone flap = 133, native bone = 18). Additional file 1: Table S1 provides the list of implant brands and models. The mean number of implants per patient was 3.8 ± 1.3 (range: 1–9) with a follow-up period of 50.0 ± 32.0 months. In 15 patients, implants were placed at the region of the irradiated bone flap. In 10 patients (20%), implants were inserted at stage I, while the remaining 30 underwent stage II implantation.

In total, 30 complications occurred (28 implants failed in 15 patients). Table 2 provides a list of complications associated with implant failure, where the main reason was lack of osseointegration (implant failure, *n* = 17) followed by peri-implantitis (implant failure, *n* = 5).

**Table 2:** Complications associated with implant failure

Reasons	Complications in patients (*N*)	Dental implants failure (*N*)
Fistula	2	5
Exposed/infected bone	1	3
Peri-implantitis	8	5
Osseointegration failure	6	17

### Survival analysis

**Fig. 3:**
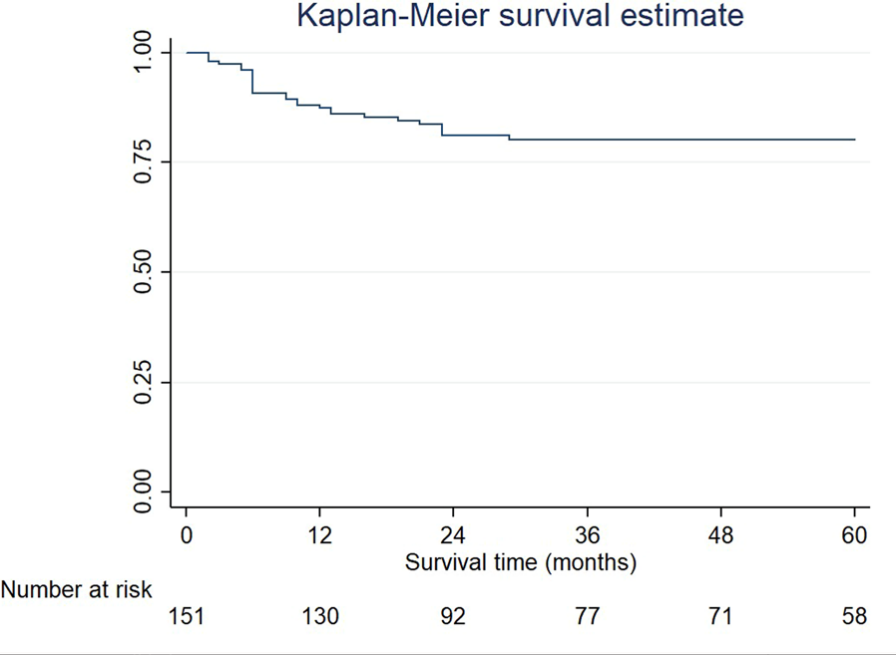
Kaplan–Meier curves of the 5-year cumulative implant survival rate

**Fig. 4:**
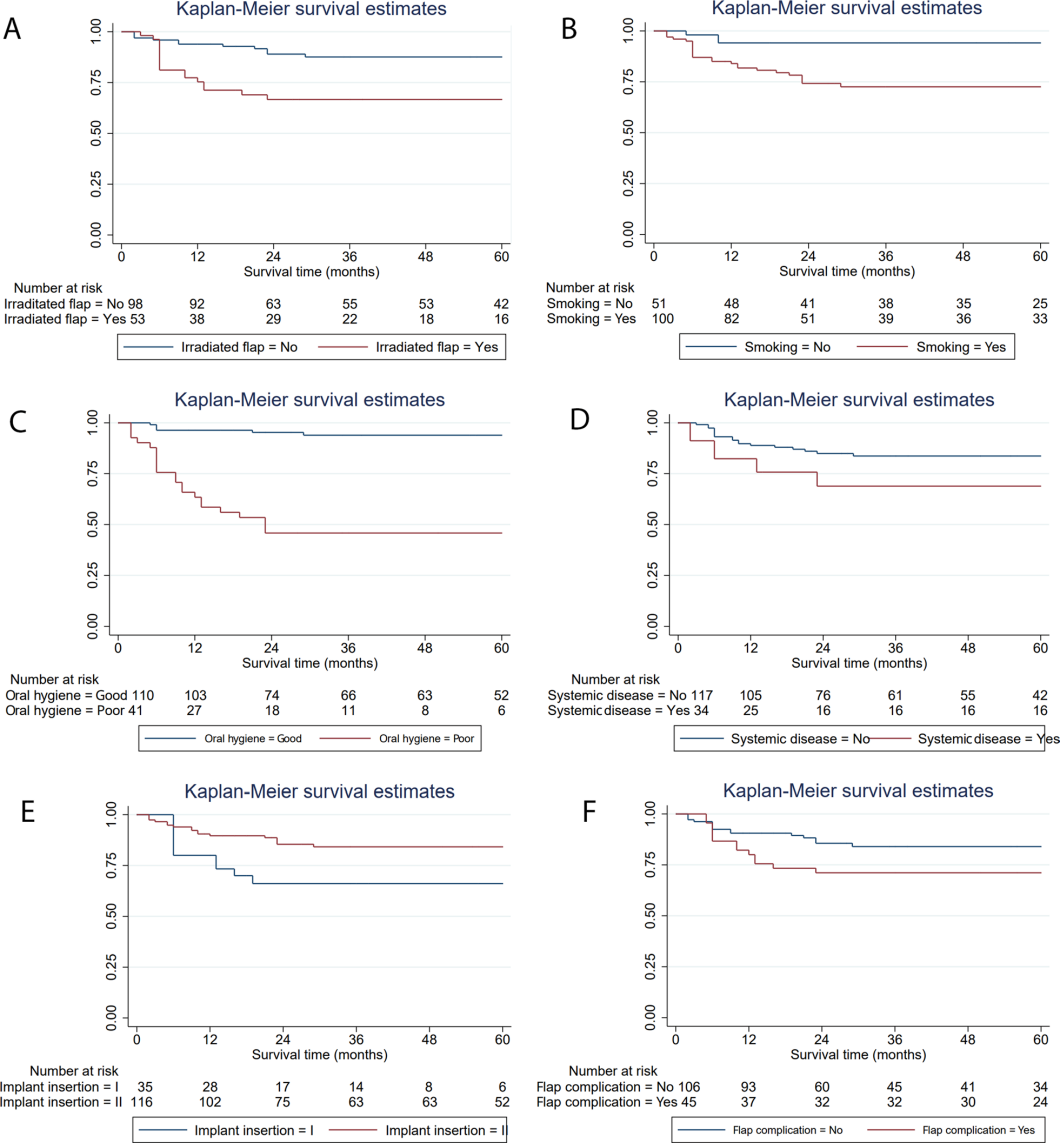
Kaplan–Meier curves of the 5-year cumulative survival rate in relation to the significant risk factors based on univariate log-rank tests. **A** Irradiated flap, **B** smoking; **C** poor oral hygiene; **D** systemic diseases; **E** implant insertion stage; **E** flap complications

Implant survival at 1-, 2- and 5-years was 96%; 87%, and 81%, respectively (Fig. 3), and the median follow-up duration was 50 months. Table 3 describes the overall implant survival rate based on the univariate analysis of the predefined patient characteristics. The following risk factors observed a statistically significant association (*p* < 0.1 in log-rank test) with implant survival: smoking (*p* = 0.004), oral hygiene (*p* = < 0.001), systemic disease (*p* = 0.052), implant insertion stage (*p* = 0.0019), irradiated flap (*p* = 0.001) and flap complications (*p* = 0.057). Figure 4 illustrates the Kaplan–Meier curves of the 5-year cumulative survival rate related to the aforementioned risk factors. Patients with a history of smoking, poor oral hygiene, systemic disease, stage I implant insertion, implant placement in the irradiated flap and flap complications were at a higher risk of implant failure.

**Table 3:** Implant survival rate based on the univariate log-rank tests

Variables	Classification	Patients (*N*)	Implants (*N*)	SR T1 (%)	SD	SR T2 (%)	SD	SR T5 (%)	SD	ST T5 (m)	SD	95% CI		*P*-value
Age	≥ 65	10	31	80.6	7.1	69.1	8.7	69.1	8.7	45.3	4.1	37.2	53.4	0.113
	< 65	30	120	89.1	2.8	86.5	3.2	83.2	3.6	51.7	1.7	48.3	55.1	
Gender	Male	26	100	89.0	3.1	85.7	3.5	83.3	3.8	51.6	1.9	47.9	55.4	0.233
	Female	14	51	84.3	5.1	82.0	5.5	71.7	7.3	47.0	3.3	40.6	53.4	
Smoking	Yes	26	100	85.0	3.6	74.2	4.6	72.5	4.8	46.8	2.3	42.3	51.3	0.004
	No	14	51	98.0	1.9	94.1	3.3	94.1	3.3	57.0	1.7	53.6	60.3	
Indication	Malignant tumor	22	83	88.0	3.6	81.6	4.3	78.1	4.8	49.2	2.3	44.6	53.8	0.335
	Benign tumor or jaw cyst	5	16	93.8	6.1	93.8	6.1	93.8	6.1	57.3	2.7	52.0	62.5	
	ORN	9	38	76.1	7.0	76.1	7.0	76.1	7.0	48.2	3.5	41.4	55.1	
	Trauma	4	14	91.7	8.0	91.7	8.0	91.7	8.0	56.6	3.3	50.2	63.0	
Site of implants	Mandible	35	130	86.2	3.0	80.0	3.6	78.9	3.7	49.6	1.8	46.0	53.2	0.257
	Maxilla	5	21	95.2	4.6	89.6	7.0	89.6	7.0	55.7	2.9	50.1	61.3	
Flap type	VFF	31	131	86.3	3.0	79.5	3.6	78.5	3.7	49.4	1.8	45.9	53.0	0.133
	VIF/VOSF	9	20	95.0	4.9	95.0	4.9	95.0	4.9	35.0	1.0	33.1	36.9	
IF	Received	15	53	75.3	5.9	66.7	6.7	66.7	6.7	43.2	3.4	36.6	49.8	0.001
	Not received	25	98	93.9	2.4	89.0	3.3	87.6	3.5	54.3	1.6	51.1	57.5	
DIIS	I stage	10	35	80.0	6.8	66.1	8.5	66.1	8.5	52.5	1.7	49.1	55.8	0.019
	II stage	30	116	90.5	2.7	85.4	3.4	84.2	3.6	43.0	4.2	34.7	51.3	
Oral hygiene	Good	27	110	96.4	1.8	95.3	2.1	93.9	2.4	57.2	1.1	55.0	59.4	0.000
	Poor	13	41	65.9	7.4	45.8	7.8	45.8	7.8	33.1	4.0	25.3	40.8	
Flap complication	Present	12	45	82.2	5.7	71.1	6.8	71.1	6.8	45.6	3.4	38.9	52.2	0.057
	Absent	28	106	90.6	2.8	85.6	3.6	84.0	3.9	52.4	1.8	48.9	56.0	
Implant location	Grafted bone	32	133	87.2	2.9	79.5	3.6	79.5	3.6	49.9	1.8	46.4	53.4	0.382
	Native bone	8	18	94.4	5.4	94.4	5.4	86.6	9.0	54.6	3.6	47.4	61.7	
Systemic disease	Present	8	34	82.4	6.5	68.9	8.2	68.9	8.2	44.5	4.1	36.4	52.6	0.052
	Absent	32	117	89.7	2.8	84.9	3.4	83.7	3.6	52.2	1.7	48.8	55.5	
Implant length	> 8 mm	34	122	88.5	2.9	82.7	3.5	81.5	3.7	50.9	1.8	47.4	54.5	0.484
	≤ 8 mm	6	29	86.2	6.4	75.6	8.0	75.6	8.0	48.3	3.9	40.7	55.9	

*SR T1* survival rate in the first year, *SR T2* survival rate in the second year, *SR T5* survival rate in the fifth year, *ST T5* survival time over the 5 years, *SD* standardized error, *IF* irradiated flap

When entering the risk factors with *p* < 0.1 into a Cox-regression model, the multivariable analyses showed that the implant survival was significantly lower in patients with systemic diseases (HR = 3.75, 95% CI 1.65–8.52; *p* = 0.002), irradiated flap (HR = 2.27, 95% CI 1.00–5.17; *p* = 0.05) and poor oral hygiene (HR = 11.67; 95% CI 4.56–29.88; *p* < 0.0001). These factors with significant association were also assessed for implant failure rate at an individual and multifactorial level to observe whether accumulated risk factors induced a higher risk of implant failure compared to individual ones. A combination of systemic disease, poor oral hygiene and irradiated flap showed the highest implant failure rate, followed by a combination of systemic disease and poor oral hygiene (Table 4).

**Table 4:** Impact of accumulated risk factors on the implant failure rate

Category	Risk factors	Patient in total (*N*)	Implants (*N*)	Failure (*N*)	Failure rate
A	Systemic disease	9	34	10	0.29
B	Oral hygiene	13	41	22	0.54
C	Irradiated flap	15	53	17	0.32
A + B		2	6	5	0.83
B + C		6	21	12	0.57
A + C		2	6	3	0.50
A + B + C		1	2	2	1.00

## Discussion

In this long-term retrospective cohort study, the 5-year cumulative implant survival rate was analyzed following OMF reconstructive surgery with a vascularized bone flap. The potential impact of risk factors on the survival rate was also assessed, which has not been comprehensively reported in the previous studies. The 5-year cumulative survival reported in this study was 81%, which was in accordance with a recent systematic review, where the authors found a survival rate of 83.4% following meta-analysis of the pooled data [12]. Additionally, comparable findings were observed with Pellegrino et al. and Alberga et al. who reported a survival rate of 86.5% and 86.4%, respectively [20, 21]. As for the 1-year survival rate, slight inconsistencies were observed. In contrast to the 1-year cumulative survival rate of 96% observed in our study, Goker et al. (85.6%) and Nguyen et al. (87.2%) found a lower survival rate, whereas Pellegrino et al. reported a higher rate (97.2%) [20, 22, 23]. These variable findings could be attributed to the different patient characteristics of the studied sample.

Based on the univariate analysis, smoking, implant placement at the region of an irradiated flap, stage I implant insertion, systemic diseases, flap complications and poor oral hygiene showed a lower implant survival rate. Furthermore, the results of multivariable Cox-regression analyses suggested an increased risk of implant failure in patients with irradiated flap, systemic diseases, and poor oral hygiene. No significant association existed between implant survival and gender, etiology, native or grafted bone-implant site, implant length, and flap type.

Fenlon et al. reported that immediate implant insertion (Pearson *χ*^2^ = 41.76.18; *p* < 0.001) and placement in the region of the irradiated flap (Pearson *χ*^2^ = 50.18; *p* < 0.001) were significantly associated with implant failure, which was consistent with the findings of the present study [24]. One could infer that the immediate implant placement and/or radiotherapy involving the flap region where the implant is placed might compromise the vitality of the graft leading to implant failure, which needs to be investigated in future studies. In addition, the importance of flap revascularization cannot be ignored. Generally, revascularization and neovascularization in the recipient bed and surrounding wound edges is sufficient to allow for pedicle division within few weeks following flap transfer [25]. However, the vascular integrity of the recipient bed is compromised in irradiated patients, which could either cause a delayed loss of the flap or negatively affect the dental implant osseointegration and survival rate. This vascular compromise is further increased in smokers, as smoking causes endothelial dysfunction and reduction in alveolar blood supply [26, 27]. Khadembaschi et al. reported a negative impact of smoking on the overall survival following implant placement in composite free flaps for reconstruction of benign and malignant head and neck pathologies [28]. As smokers are at a higher risk of post-operative infection, marginal bone loss and implant failure, which has been confirmed by various studies [29]. Previous evidence suggests only a few studies assessing the association between oral hygiene and dental implant survival rate following jaw reconstruction. The lower survival rate in patients with poor oral hygiene could be attributed to the fact that plaque accumulation might induce an inflammatory reaction leading to secondary implant failure due to peri-implantitis [30, 31].

Native bone had a higher implant survival rate compared to the grafted bone, which was consistent with Ch'Ng et al. and Jacobsen et al.’s findings, who also reported a higher implant failure placed in bone flap compared to the native jaw [32, 33]. The most likely reason could be the impact of radiotherapy, poor oral hygiene and/or smoking. However, the limited number of implants placed in the native bone did not allow isolation of specific risk factors, thereby, requiring further studies with a larger sample size to assess the reasons for implant failure. Additionally, the majority of patients in the present study underwent reconstruction with fibular flap, which is mainly composed of dense cortical bone and its thickness has been known to significantly reduce at a long-term follow-up which might also impact the implant survival [34]. Hence, requiring further investigations for assessing survival outcome based on bone thickness, especially if implants are placed immediately at the time of reconstruction.

A relatively lower survival rate of implants was observed in patients with a malignant tumor and osteoradionecrosis, which could have been due to the administration of radiotherapy in a majority of the patients [35]. Previous studies have also observed a detrimental impact of radiotherapy at both reconstructed and native bone sites, which leads to a higher implant failure and patients suffer from an increased risk of post-implant surgery complications [36]. Therefore, the key for having a high implant survival rate following reconstructive surgery is to devise a patient-specific treatment plan considering the influence of the aforementioned risk factors at both individual and accumulative levels. Recent improvements in implant designs, surface modifications and shifts in treatment strategies have improved implant osseointegration and long-term survival rate following surgical reconstruction and radiotherapy. Furthermore, the application of three-dimensional planning and computer-guided implant surgery should also be taken into consideration for increasing the implant survival rate, as it offers several advantages over conventional approaches, such as improved accuracy of dental implant placement, maintenance the periosteal irrigation and possibility of performing a flapless procedure [37, 38].

The study had certain limitations. Firstly, a historical bias existed due to the inclusion of both freehand and CAS-based techniques with the presence of different adjuvant chemo-radiotherapeutic strategies. Secondly, the assessment of certain individual risk factors and accumulated risk of multiple factors on implant failure rate suffered from a limited sample size with a lack of statistical power, which should be interpreted with caution. Finally, the study involved a consecutive group of patients rather than one specific patient population. Future studies with a larger and standardized sample size are required to reach a definitive conclusion. Despite the limitations, the study provided a comprehensive report of the risk factors associated with implant survival which could allow improving the decision-making process and treatment planning in patients undergoing OMF reconstructive and implant surgery.

## Conclusions

The cumulative implant survival rate was highest at 1st year, followed by 2nd and 5th year, indicating that the risk of implant failure increased over time. Risk indicators that seem to be detrimental to long-term survival include poor oral hygiene, irradiated flap and systemic diseases. Prospective studies are warranted to further elucidate the factors contributing towards implant failure, to allow for optimal patient-specific delivery of care while striving for a long-term positive outcome.

## Supplementary Information

**Additional file 1: Table S1.** Implants brand and model.

## Data Availability

The datasets used and/or analyzed during the current study are available from the corresponding author on reasonable request.

## Abbreviations

OMF: Oral and maxillofacial
VFF: Vascularized fibula flap
DCIA: Deep circumflex iliac artery flap
VOSF: Vascularized osteomyocutaneous scapular flap
STROBE: Strengthening the Reporting of Observational Studies in Epidemiology
CBCT: Cone-beam computed tomography
CAS: Computer-assisted surgery
STL: Standard Tessellation Language
HR: Hazard ratio
DIIS: Dental implant insertion stage
ORN: Osteoradionecrosis
