# Supplementary material for: Long-term survival of implant-based oral rehabilitation following maxillofacial reconstruction with vascularized bone flap
Source: Int J Implant Dent. 2022 Apr 5;8:15. doi: 10.1186/s40729-022-00413-7 (PMC8980171; doi:10.1186/s40729-022-00413-7)
Supplement: Supplementary file 1 — Additional file 1: Table S1. Implants brand and model. [file 40729_2022_413_MOESM1_ESM.docx]

**Table S1. Implants brand and model.**

| ID | Implant brand | Implants (N) |
| --- | --- | --- |
| 1 | Nobel Brånemark System Mk III | 5 |
| 2 | NobelActive RP | 4 |
| 3 | Astra Tech Implant System for OsseoSpeed TX | 3 |
| 4 | Astra Tech Implant System for OsseoSpeed TX | 4 |
| 5 | NobelActive RP | 4 |
| 6 | Ankylos | 4 |
| 7 | Straumann BLT | 4 |
| 8 | Astra Tech Implant System for OsseoSpeed TX | 2 |
| 9 | NobelActive RP | 4 |
| 10 | NobelActive RP | 3 |
| 11 | Ankylos | 4 |
| 12 | Bicon | 3 |
| 13 | Bicon | 9 |
| 14 | Bicon | 4 |
| 15 | Bicon | 4 |
| 16 | NobelActive RP | 6 |
| 17 | Bicon | 4 |
| 18 | Bicon | 4 |
| 19 | NobelActive RP | 4 |
| 20 | NobelActive RP | 3 |
| 21 | Astra Tech Implant System for OsseoSpeed TX | 4 |
| 22 | NobelParallel CC RP | 4 |
| 23 | NobelParallel CC RP | 2 |
| 24 | NobelParallel CC RP | 3 |
| 25 | Visafix | 3 |
| 26 | Straumann BLT | 2 |
| 27 | NobelReplace CC | 4 |
| 28 | NobelReplace CC | 4 |
| 29 | NobelParallel CC RP | 4 |
| 30 | NobelParallel CC RP | 7 |
| 31 | NobelReplace CC | 4 |
| 32 | NobelParallel CC RP | 3 |
| 33 | NobelReplace CC | 3 |
| 34 | Astra Tech Implant System for OsseoSpeed TX | 2 |
| 35 | Astra Tech Implant System for OsseoSpeed TX | 4 |
| 36 | NobelParallel CC RP | 4 |
| 37 | Astra Tech Implant System for OsseoSpeed TX | 3 |
| 38 | Astra Tech Implant System for OsseoSpeed TX | 4 |
| 39 | NobelParallel CC RP | 2 |
| 40 | Straumann BLT | 3 |
| Total |  | 151 |
